# Treatment of pancoast tumors from the surgeons prospective: re-appraisal of the anterior-manubrial sternal approach

**DOI:** 10.1186/1749-8090-5-102

**Published:** 2010-11-04

**Authors:** Haralabos Parissis, Vincent Young

**Affiliations:** 1 Cardiothoracic Dept, Royal Victoria Hospital, Belfast, Northern Ireland; 2 Cardiothoracic Dept, St James Hospital, Dublin 8, Dublin, Ireland

## Abstract

Pancoast tumours are now amenable to multimodality treatment with an acceptable survival. This is because trimodality treatment improves tumor sterilization and hence outcome. Moreover the development of an anterior approach to access the tumor, further improved the technical challenges for a sound resection.

The Anterior-manubrial sternal approach was described more than a decade ago and although this method facilitates better exposure of the extreme apex of the lung, brachial plexus and subclavian vessels, its popularity has not reached high levels. We felt that by re-addressing this topic we would stimulate reconsideration of the anterior approach.

## Introduction

Pancoast syndrome is due to lesions extending to the superior thoracic inlet. Specific symtomatology mainly due to brachial plexus invasion accounts for the majority of those cases [1-3].

Pancoast tumour is a tumour of the apex of the lung with no intervening lung tissue between tumour and chest wall. Subsequently, there is an involvement of structures of the apical chest wall above the level of the second rib. Almost half of the treated cancers are squamous cell carcinomas (45-50%), while the rest are either adenocarcinomas (36-38%) or undifferentiated large-cell carcinomas (11-13%). The tumour rapidly involves the structures of the thoracic inlet & the root of neck. Due to its localization in the apex of the lung, invasion of the lower part of the brachial plexus, first ribs, vertebrae, subclavian vessels or stellate ganglion, occurs [4]. The classical Pancoast presentation, with shoulder pain radiating to the ulnar side of the arm and the hand, is presented in 55 to 60% of the patients. Pain at the ulnar aspect of the forearm and hand is consistent with T1 involvement; furthermore symptomatology along the intrinsic hand muscles suggests the C8 root or lower trunk tumor deposits. Horners syndrome is reported in up to 30% of the cases.

Although those tumours represent a wide range of stage IIB to stage IV disease, [IIB (25-27%), stage IIIA (6-8%), stage IIIB (40-42%) and stage IV (21-23%)] it is the T3, T4, N0-N1 subgroup of this spectrum that could be amenable to surgical intervention [5]. This subgroup of patients (less than 5% of Bronchogenic Carcinomas) however, is difficult to be treated surgically due to the location of the tumour and the complex anatomy of the area involved [6]. Historically, Pancoast tumors have been associated with high rates of incomplete resection, local recurrence, and death.

Pancoast tumours were thought to be located posteriorly and early attempts to resect those tumors were approached solely from the back. A percentage of these lesions might also be located at the front, with vascular rather than neuro-vertebral involvement. Various reports suggested spinal involvement in 15%, brachial plexus in 15% and subclavian vessels in 6% of the cases [7]. Therefore surgeons treating these cancers should be able to be familiar and adapt with the various approaches.

An understanding of the posterior location of neural structures and somewhat anterior location of vascular structures is important for adequate operative planning.

It is worth noted that the popularity of this approach has not reached high levels of acceptance in Britain (First National Thoracic Surgery Activity & outcomes Report from the Society for Cardiothoracic Surgery in Great Britain & Ireland/2008). Our experience consists of a handful of cases therefore with the present article we attempt to elaborate on the anatomy, initial assessment, and surgical approaches with an emphasis on the modified anterior approach for this form of cancer.

### The evolution of the treatment

For more than 40 years the treatment of Pancoast tumors has centered on a bimodality regimen consisting of preoperative external beam radiotherapy followed by surgery. Trimodality treatment however with the addition of platinum based chemotherapy regimes has become currently the standard treatment, in order to achieve additive anti-tumour effects (chemotherapy as radiation sensitizer). According to Wright et al [8] induction Chemoradiotherapy (CT/RT) can be administered with low morbidity, a higher complete resection rate, a high pathologic response rate, a reduced locoregional recurrence rate and improved survival. Further improvement in radiotherapy with the advent of 3-dimensional conformal radiotherapy, the total radiation dose that could be safely delivered was not anymore constrained by dose-limiting toxicities upon the nearby organs.

Careful patient selection for trimodality treatment, on the basis of staging and comorbidity, is of vital importance in the treatment of Pancoast tumours. Nevertheless only 30% of M0 patients with Pancoast tumors were eligible for combined treatment according to Pourel et al [9].

Not only operapability (patient fitness to surgery) but also ability to resect the tumour is of a major importance bearing in mind the difficulty of access, the crowded anatomy of this region and the tendency of the tumors in this area to involve important adjacent structures. As per the same group [9], following CT/RT, 67% of the patients were amenable to thoracotomy. The resection rate, which had remained unchanged at approximately 50% for almost 40 years with conventional preoperative radiotherapy, was improved to above 70% in SWOG [10] and JCOG [11] studies.

Preoperative radiotherapy was part of the standard treatment, but a recent prospective phase II study (Southwest Oncology Group 9416, INT 0160), [10] suggests that preoperative concurrent CT/RT (platinum-based chemotherapy and 45 Gy of radiotherapy) improves the rate of complete resection, local recurrence, and intermediate-term survival.

Like wise, the Japan Clinical Oncology Group JCOG trial 9806 [11] in a prospective report concluded along similar lines. Furthermore, Kwong et al [12] reported that high dose radiotherapy targeting up to 60 Gy (rather than 45 Gy) can be given in the neoadjuvant setting; it is successfully tolerated and associated with improved resection rate.

### Surgical considerations

The limited access and poor visualization of the thoracic inlet is due to: 1) the unique course of the upper ribs downwards and outwards that render the neurovascular bundle inaccessible to posterior approaches, 2) the musculature of the area and also 3) the overlapping bulky pectoral-shoulder girdle with the clavicle and the manubrium to further restrict access from the neck. These anatomical idiosyncrasies create a hostile but challenging environment for the thoracic surgeon.

The main goal for cure is to achieve local control of the disease and aim for relapse-free, metastasis-free outcome. Local control is obtained by removing the upper lobe, chest wall and invaded structures (subclavian artery or vertebra), aiming for R0 resection margins. Radically resected cases yield better survival whereas R1 resections are associated with high incidence of local and distal recurrences.

Involvement of the vertebral body or brachial plexus, once considered unresectable is nowadays amenable to advanced techniques of spinal reconstruction and should be planned jointly with a spine neurosurgeon.

Finally, according to recent reports [10,11] the rate of R0 resection could be above 85%, with the use of trimodality protocols.

Contraindications for surgery would be due to metastasis, invasion of the brachial plexus above C7 & invasion of the spinal canal. Resection of the T1 nerve root is usually well tolerated, but removal of the C8 root or lower trunk of the brachial plexus leads to loss of hand and arm function. N2 disease, is a relative contraindication and some groups enroll those patients after extended hilar radiation.

As per JCOG [11] rib involvement occurs in 77.2% of the patients (usually 3 ribs or more), vertebra involvement in 10.5% of the patients, and major vessels in 5.3%. T1 involvement is the commonest root involved in up to 85% of the cases.

### Downstaging

According to Wright et al [13] marked difference in pathologic response based on the induction therapy is favoring CT/RT.

Surgical resection of Pancoast tumors after neoadjuvant high-dose CT/RT was carried out in 40.5% of patients according to Kwong et al [12].

Pathological downstaging although it does not correlate with the radiological appearance [10] is reported to be impressively above 30% in various series.

As per Pourel et al [9], pathological complete response was observed in 39.5% of the patients, necrosis of tumoral tissues between 50% and 95% in 22.5% and less than 50% in 38% of the patients. Along the same lines, JCOG reported [11] pathologic downstaging of the tumor in 40% of the patients; No residual viable tumor cells in the resected specimens, was achieved in 16% of the treated patients. Finally SWOG [10] summarized that pathologic no residual microscopic tumor was seen in one third of the resected specimens and minimal microscopic residual (few scattered tumor foci within a mostly necrotic or fibrotic mass), was observed in one third of the resected specimens.

### Surgical Approaches

Posterior approach (Paulson)/posterolateral-paravertebral thoracotomy: This is an extension of the conventional postero-lateral thoracotomy; the incision is extending around the tip of the scapula, then it continuous upwards and further midway between the posterior edge of the scapula and the spinous processes, up to the level of C7. By taking the scapula of the chest wall this incision allows good exposure of the posterior chest wall, including the transverse processes, the vertebrae and the roots of the thoracic nerves and the plexus [14]. Never the less the exposure of the neurovascular structures are limited. This is due to the fact that brachial plexus and vascular structures often lie above the tumor mass and access to such structures, is significantly limited using approaches from below.

According to Vanakesa et al [15], Posterior approach, does not provide adequate access to the many important structures which may be involved by apical chest tumors of bronchogenic origin. This restricted access may be one of the reasons for the high rate of incomplete resections [16] and high surgical morbidity and mortality using this approach [13].

The anterior-cervical entry [17] proved to be the answer to the problem of limited exposure. It appears to be the optimal approach to anterior lung apex or first rib lesions [18].

**Figure 1:**
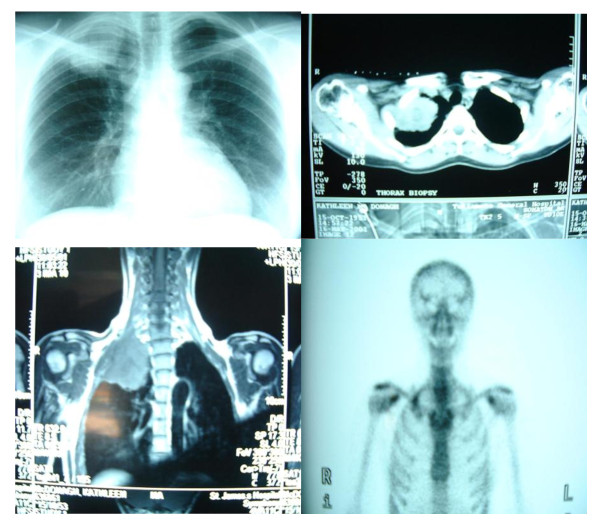
**CXR, CT Chest imaging, MRI and bone scan of a Pancoast tumor of a 47 yrs old female, Ex smoker (25 cigs per day up to 13 years ago)**. Six weeks history of shoulder pain radiating to the median aspect of the right arm. CXR mass at apex of right chest. Percutanteous Biopsy NSCLC. PMH: Hysterectomy for Ca cervix 1996 - no evidence of recurrence. Clinical examination fullness in right supra-clavicular fossa

We would facilitate a case like the one presented in Figure 1 by using an Anterior-manubrial-sternal approach for access.

**Figure 2:**
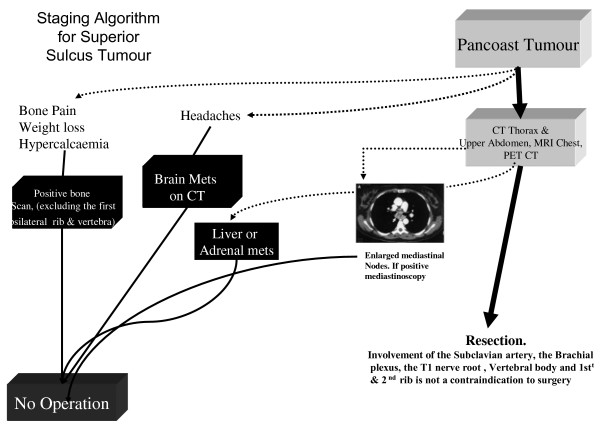
**Staging algorithm for patients prior to resection of a Pancoast Tumor**. MRI of the thoracic inlet may yield further information's on the status of vertebra involvement

Accurate and thorough staging & re-staging (Radiological response is defined according to the RECIST criteria [19]) following neo-adjuvant treatment is necessary prior to surgery (see Figure 2) and typically includes CT-PET and magnetic resonance imaging (Contrast-enhanced MRI of Chest and Brain). MRA is a noninvasive diagnostic method complementary to MR imaging for detecting vascular involvement in bronchogenic carcinoma with Pancoast syndrome [20].

**Figure 3:**
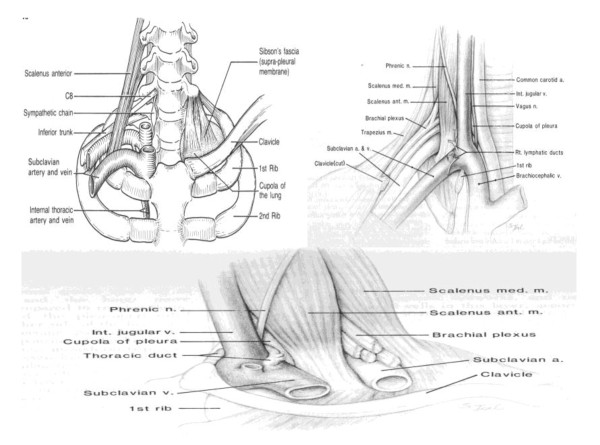
**Root of neck anatomy, depicting carefully the relationship of the most important neurovascular structures to the scalene musculature and the first rib**.

Root of neck anatomy as in Figure 3 is depicting carefully the relationship of the most important neurovascular structures to the scalene musculature and the first rib. The anterior and middle scalene muscles are attached to the first rib and can be used as landmarks: in front of the anterior scalene muscle situated the subclavian and internal jugular veins and the sternocleidomastoid and omohyoid muscles.

The subclavian artery, the trunks of the brachial plexus, and the phrenic nerve are emerging above the lateral part of the first rib between the anterior and middle scalene muscles. The nerve roots of the brachial plexus, the stellate ganglion, and the vertebral column are situated behind the middle scalene muscle.

### The Surgical steps (Figure 4)

**Figure 4:**
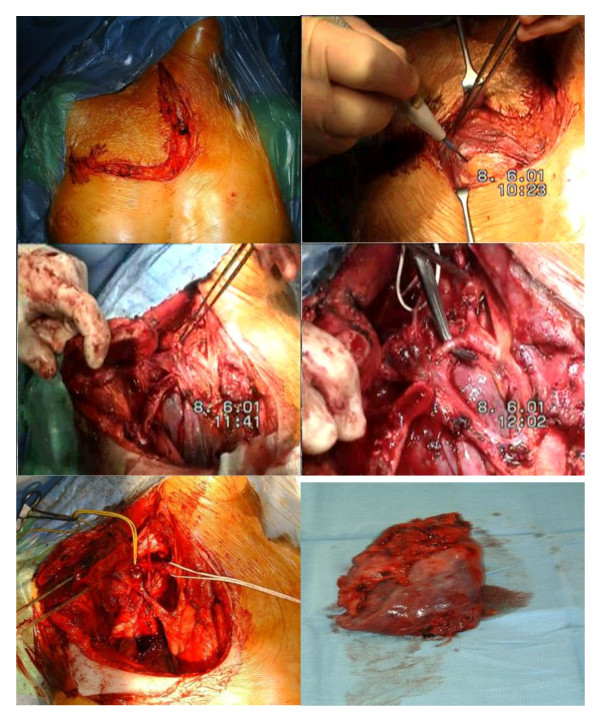
**Step by step resection of a Pancoast tumor through an Antero-cervical approach**. Incision at the anterior edge of Sterno-cleido-mastoid (a). Division of the upper sternum extended into 2^nd^ intercostal space(b). Mobilisation-Excision of supraclavicular fat pad (c). Exposure of the structures at the thoracic inlet by dividing the subclavius, omohyoid with preservation of the accessory nerve. Division of the Scalenus anterior with preservation of the phrenic nerve (d) & (e). Right upper Lobectomy (f): can be performed through the neck incision or a posterolateral thoracotomy.

We favor a modified Dartevelle approach [17] an L shaped incision at the anterior edge of Sterno-cleido-mastoid (2). Division of the upper sternum extended into 2^nd ^intercostal space. This is a modified access something between Grunenwald [21] and Klima et al [22] approach. Grunenwald has described a transmanubrial approach, which avoids division of the clavicle. Klima and colleagues suggested extending the L-shaped section of the manubrium down to the first intercostal space. We prefer to divide the sternum down to the angle of Luis and then extend the incision horizontally along the 2^nd ^intercostal space, thus allowing the surgeon to lift the clavicle, subclavian muscle, and transected part of the manubrium and superior body of the sternum without dividing the first costal cartilage and ligament. The internal mammary artery is encountered and divided during the horizontal intercostal incision.

Mobilisation & excision of the supraclavicular fat pad (3), allows exposure of the structures at the thoracic inlet; further division of the subclavius, omohyoid with preservation of the accessory nerve is carried out.

The distal part of the jugular veins is divided to expose the subclavian and innominate veins. If the subclavian vein is affected then it is resected. Following this, the scalenus anterior muscle is divided by taking care to preserve the phrenic nerve (4) & (5). The subclavian artery is mobilized by, dividing most of its branches. Care is taken to preserve the vertebral artery and resection of the vessel is done only if it is involved with the tumor and no substantial extracranial occlusive disease can be detected on preoperative Doppler ultrasound.

If the subclavian artery is taken up by tumor, the affected portion is resected and reconstructed, usually with a 6-8 mm PTFE vascular graft. Small dose of heparin is usually administered during vascular clamping.

Following anterior traction of the subclavian artery, the scalenus medius muscle comes into good view. The muscle is divided above its insertion on the first rib, giving access to the branchial plexus. Familiarity with the anatomy of the plexus is important. At this stage, the anterior surface of the vertebral bodies of C7 and T1 are in view. The sympathetic chain and stellate ganglion are lying in front of the anterior surface of the vertebral bodies of C7 and T1. The C8 and T1 nerve roots are visualized and dissected medially up to the lower trunk of the brachial plexus. The C8 nerve component of the plexus is preserved if possible, for better functional outcome of the upper limp.

Care is taken then, to access tumor invasion and plan with the neurosurgeon the "spinal component" of the operation.

Chest wall resection is carried out by dividing the first 2-3 ribs at the sternal - costochondral junction following by disarticulation of the ribs from the transverse processes at the back. The last part of the resection consists of the upper Lobectomy (6). The access to perform a lobectomy and mediastinal lymph node clearance through the anterior incision is usually limited, therefore like others [23] we perform a traditional posterolateral thoracotomy through the 5^th ^IC space. Routine coverage of the bronchial stump with an intercostal or serratus muscle flap is advocated by some groups [12] to counteract any potential damage on the stump from the neoadjuvant radiation. Chest wall reconstruction may be necessary in up to 40% of the cases [23].

For Pancoast carcinomas affecting the spine, a posterior midline approach can be added by a neurosurgeon, for multilevel unilateral laminectomy [24], nerve root division inside the spinal canal, and vertebral body division along the midline. The tumor then is removed en bloc with the lung, ribs, and vessels through the posterior incision. Fixation of the spine is mandatory.

### The advantages of the Anterior-Cervical approach

According to Machiarini et al [25] one of the major advances in the treatment of Pancoast tumors has been the introduction of anterior approaches for resection. These approaches increase the likelihood of complete resection and permit resection of tumors that were previously considered technically unresectable [26].

Furthermore anterior approach facilitates:

1) Direct visualization of major structures (eg. Subclavian artery, superior vena cava) thus allowing control and elective sacrifice of the artery if necessary and reconstruct directly to a safe outcome.

2) Excellent exposure of the brachial plexus, sympathetic chain, and stellate ganglion.

3) Freedom to carry out hemi-vertebrectomy if the anterior body of the vertebrae are involved.

4) Resection of the lower parts of the Brachial plexus, especially of the C8, T1 roots; however T1 root resection results in diffuse weakness of the intrinsic muscles of the hand, whereas resection of the C8 nerve root of the lower trunk of the brachial plexus results in permanent paralysis of the hand muscles

5) Optimal access, for resection of the chest wall

6) Oncological clearance of the structures of the Thoracic inlet, because the tumor is the last to be encountered.

7) Lower morbidity than the posterior approach

Moreover as per Vanakesa et al [15] the cervical-trans sternal approach has several advantages, chiefly that of avoiding disfigurement and loss of function of the pectoral girdle, whilst providing excellent exposure of the brachial plexus, sympathetic chain, and stellate ganglion. Such an approach results in a short postoperative stay (3-6 days), and yet allows extension as per Grunenwald [21], or by a high, anterior thoracotomy if necessary.

### Disadvantages of the Anterior-Cervical approach

Removal of transverse processes and the head of the ribs in order to disarticulate them, could be difficult with the anterior access; furthermore more posterior seated tumors with vertebra involvement may require a complimentary posterior incision.

There are concerns about functional and aesthetic results with the transclavicular approach, which includes removal of the medial half of the clavicle.

Finally, the need to perform an additional posterolateral thoracotomy for the lobectomy and mediastinal node clearance could be seen as a factor that negates any advantage of the routine use of the anterior-manubrial sternal approach.

## Results

Unfavorable outcome is due to incomplete resection and life-threatening complications.

Current reports are quoting perioperative mortality not higher than for any other lung resection [10,11].

Adverse prognostic factors, are including the presence of mediastinal nodal metastases (N2 disease), spine or subclavian-vessel involvement (T4 disease), and limited resection (R1 or R2) [27-29]. Along similar lines, Ginsberg et al. [30] found Horner's syndrome, N2/N3 disease, T4 disease and incomplete resection, in general, to be adverse prognostic factors. Okubo and associates [16] found that incomplete resection particularly tumour invasion to the brachial plexus, influenced the prognosis.

### Recurrence

With bimodality regimes the local recurrence rates were reported to be above 70% [7,13]. Despite the advent in treatment regimes, local recurrence still occurs in about 40% of the patients [29]; it is expected that local recurrence rate is higher in patients with T4 disease because complete resection can be achieved in less than half of the patients with c-T4 disease [11]. More specifically [27] complete resection rate was achieved in only 64% of tumour stage T3 and nodal stage N 0 and 39% of T4N0 tumours. It is apparent however, that locoregional relapse is predominant in R1-2 resections, whereas distant recurrence is frequent in R0 resections.

One would expect that a shift in the trend of clinical recurrences towards distant metastasis is to be currently expected because of the fact that trimodality treatment facilitates better R0 resection. As per Pourel et al [9] the most frequent site of relapse was distant metastasis in 66% of the patients, (mainly brain) with the locoregional recurrence rate been 18%. Likewise King et al [13] reported brain metastasis in 25% and local recurrence rate in 19% of the cases. A small series that had bimodality treatment however had an incidence of locoregional recurrence of 17.2% [8]

Survival has been extensively reviewed by Attar et al [31]. Overall survival at 5 years after surgery was 46% for T3N0, 13% for T4N0, and 0% for lesions with N2 disease [27]. Particularly noteworthy [11] was the reproducibility of the favorable survival data, with a 5-year overall survival rate of 44% in the United States trial (SWOG) and 56% in JCOG trial, which were clearly superior to the historical value of 30%.

## Future

In the future new neoadjuvant regimes including aggressive protocols of accelerated radiotherapy would potentially increase the pool of surgical candidates from patients diagnosed with a Pancoast tumor (currently 23% of the patients as per Kappers et al [7]). However, several questions still remain unresolved:

1) The role of PET-CT in restaging tumors (eg. The role of "late wash out" images in differentiating between inflammation and residual tumor) following neoadjuvant treatment; Schmuecking et al. [32] have shown that metabolic response after induction CT/RT evaluated within 1 week following its completion, is highly predictive of pathological response.

2) What is the significance and implications of ipsilateral supraclavicular lymph node disease: The argument being that these nodes are in close vicinity of the tumour and therefore could have the characteristics of the biological behaviour of "N1 disease".

3) Recruiting patients with N2 disease: The argument being that inclusion of the hilar and mediastinal nodes in the irradiation field promotes downstaging. Kwong et al. [12] did not exclude patients with positive mediastinal nodes from trimodality treatment and found no difference in survival. In most papers, however, results of patients with persistent N2 disease turned out to be clearly inferior to those of patients with N0/1 only. On the other hand, no clinical trial has yet compared various trimodality treatment regimes for patients with N2 disease.

4) The role of prophylactic cranial irradiation: Due to good locoregional control with trimodality treatment, distant metastases now represent the most common site of failure. Furthermore, the incidence of brain metastasis as a first site of recurrence in Pancoast tumour is between 15-30% [23,33]. The negative impact of brain metastasis on survival has to be weighed against the risks benefits ration of the impact of prophylaxis with radiation to the brain

5) The role of high dose of RT (up to 60 Gy): Are there specific subgroups (eg. for patients with clinical T4 disease complete resection is feasible in less than 50% of the cases) that they would benefit

6) The role of Adjuvant postoperative chemotherapy: distant metastases now represent the most common site of failure following treatment for Pancoast tumors therefore preventing distant metastasis has now become the challenge in the treatment of these patients. Large randomized trials concluded a 5--15% survival benefit at 5 years of adjuvant chemotherapy in patients with radically resected stages I--IIIA NSCLC [34,35] However, many patients with Pancoast tumors may not tolerate more extensive treatment. Moreover Martinod et al. [36] reported that preoperative radiotherapy significantly improved the 5-year survival for stage IIB--IIIA, while postoperative radiotherapy and chemotherapy did not significantly alter survival. The survival benefit with the use of the anterior approach for the same stage of Pancoast tumors versus the posterior approach also remains to be seen.

## Conclusion

Pancoast tumors represent a small percentage of Lung cancer population (1-5%). Due to poor performance status and/or advanced tumor stages, only 30-40% [7,13] of those patients are eligible to be enrolled in multimodality protocols of treatment.

Careful patient selection and adherence to protocols enables Clinical groups to get an impression of the efficacy of an intervention and to compare results between studies.

No single surgical approach however, provides the best access to all heterogeneous tumors of the thoracic inlet. What probably provides the most favorable outcome would be a team approach, where the thoracic surgeon coordinates with an experience neuro-spinal surgeon, in a background of limited disease that is responding well to neoadjuvant chemoradiotherapy.

Finally, the thoracic surgeon must be familiar with the potential advantages that the anterior approach offers under given circumstances. This knowledge enables the thoracic surgeon to explore new avenues and exciting challenges. Darteville's approach and the various modifications are technically demanding, however, once the anatomy has been appreciated, direct visualization of the major structures of the Thoracic inlet aids to facilitate complete oncological clearance. Finally, whether the anterior approach results in less locoregional recurrences and possibly better 5 year survival, remains to be tested.

## Competing interests

The authors declare that they have no competing interests.

## Authors' contributions

HP conceived of the study and wrote the manuscript. VY overlooked the progress of the manuscript and advised on valuable points. All authors read and approved the final manuscript.
